# Multi-level quality assessment of United States COVID-19 epidemiological surveillance

**DOI:** 10.1371/journal.pone.0319263

**Published:** 2025-02-21

**Authors:** Megan S. Irgens, Riley M. O’Neill, John M. Ruiz

**Affiliations:** 1 Juvenile Justice Behavioral Health Lab, Division of Infant, Child, and Adolescent Psychiatry, Zuckerberg San Francisco General Hospital, University of California San Francisco, San Francisco, California, United States of America; 2 Department of Psychology, University of Arizona, Tucson, Arizona, United States of America; Kisumu County, KENYA

## Abstract

Although the coronavirus disease 2019 (COVID-19) tasked United States (U.S.) health departments with tracking and informing disease mitigation efforts, there has been no national assessment of public COVID-19 data quality. The current study aimed to illustrate U.S. COVID-19 reporting quality by examining epidemiological surveillance from U.S. health departments in 50 states and the District of Columbia between July and August 2020 along five central quality dimensions: timeliness, reliability, completeness, accuracy, and accessibility. A follow-up assessment was conducted 19 months later for a subset of states. Results broadly demonstrated that the quality of COVID-19 data reporting was significantly heterogenous. States varied in timeliness of initial data reports (median: 3/2/2020, minimum: 9/29/2019, maximum: 4/20/2020). Most states (56.8%) did not indicate sources of data for their official published reports. In assessing for 10 standard variables from the Centers for Disease Control and Prevention (CDC), states widely differed in granularity of reported variables and no state health department reported data for all CDC surveillance variables (e.g., race, ethnicity, sex). Most same-day comparisons of state-specific counts reported by the CDC differed from respective state health departments (cases: 84.3% differed; deaths: 68.6% differed). Follow-up examination indicated improvements in reliability, accuracy, and accessibility, but issues in data source verification and completeness persisted. Authors conclude with recommendations to improve disease mitigation efficiency and improve public trust in health protection efforts: establish formal reporting guidelines; standardize operational definitions for key disease variables; and, require health departments to publicly identify sources of reported data.

## Introduction

The coronavirus disease 2019 (COVID-19) became the third leading cause of death worldwide as of 2021, infecting more than 375 million people and accounting for over 5 million deaths [1]. The emergence of multiple COVID-19 variants has further contributed to containment challenges and societal anxiety regarding public health mitigation capabilities. Previous research pertaining to the United States (U.S.) has demonstrated that in September 2021, COVID-19 related-deaths surpassed the total deaths from the 1913 Spanish flu pandemic and that COVID-19 had reduced contemporary life expectancy by 1.5 years [2]. Enforcement of evidence-based behavioral recommendations for disease mitigation (e.g., social distancing, face masks) has been significantly heterogenous across the U.S. by region and date [3]. This heterogeneity has brought attention to the efforts of accurately tracking and disseminating disease information.

Accurately tracking infection trends is a necessary prerequisite to successfully strategizing and deploying effective disease mitigation efforts. The nation’s public health recording of cases has been mired in a series of varying decision processes at the local and regional levels regarding what constitutes a case, how and when that information is communicated to public health offices, and the aggregation of potentially heterogeneous data. This multilevel discordance in procedures for tracking and reporting infection trends may be associated with several potential consequences, including poor disease surveillance, inefficient resource allocation for disease mitigation, unchecked infection outbreaks, and an erosion of public trust in the accuracy of COVID-19 related information.

### Quality of data and impact on apparent reliability

Theoretical and empirical support demonstrates the quality and reliability of disease data are critical determinants of individuals’ willingness to engage in disease mitigation behaviors [4–6]. The Health Belief Model, a prominent conceptual framework for public health research regarding uptake of health-protective behaviors, identifies individuals’ belief they are susceptible to contracting a disease as a critical determinant of their likelihood to take actions they believe will effectively reduce their risks [4]. Relatedly, previous studies during other infectious disease outbreaks (e.g., Middle East respiratory syndrome; vector-borne diseases [malaria, dengue fever]) have demonstrated robust associations between individuals’ reported perceptions of disease risk from public epidemiological data and likelihood to adopt behaviors supported for mitigation of respective diseases [5–7].

Ning et al. [7] demonstrated perception of COVID-19 disease risk and trust in governmental communication of disease data significantly predicted Chinese citizens’ likelihood to adopt protective behaviors against COVID-19 transmission throughout January and February 2020. Knowledge of local disease prevalence, transmission trends, and reported outcomes (e.g., hospitalization, deaths) are forms of disease information that individuals seek from local, statewide, and national communication to guide their understanding of personal disease risk [8]. These findings underscore the importance of the quality and reliability of official communication of disease data when orchestrating responses to public health crises, such as the COVID-19 pandemic. Recognition of data quality’s importance is also reflected in the U.S. Department of Human Health and Services Pandemic Plan 2017 Update [9], which included several criteria supporting the improvement of accuracy and fidelity in the reporting of epidemiological data quality at state and federal levels.

### Assessing quality of data

Although there is no gold standard for assessing quality of public health data, timeliness, accuracy, and completeness are constructs most often used to describe these data [10]. These constructs describe the frequency of database updates, accuracy of the data, and robustness of the dataset. Publicly available data, like those reported on health department websites and the Centers for Disease Control and Prevention (CDC), require additional assessment of data quality due to being open access. Due to the overlap in constructs between previous studies assessing the quality of public health data and open data source studies, the current study utilized an evaluation framework for open data sources that includes the traditional constructs of timeliness, accuracy, and completeness in addition to expanded assessment of dataset accessibility and reliability [10,11]. Accessibility refers to the feasibility of using the data as a public consumer (e.g., outside researcher, citizen scientists), whereas reliability describes the extent to which the sources of reported data are identified and trusted.

### Current study

Since the beginning of the COVID-19 pandemic in January 2020 through December 2023, there have been over 103 million cases of COVID-19 and over 1 million deaths from COVID-19 in the U.S [1]. With several federal and state outlets for people in the U.S. to obtain information to help evaluate disease risk, it is imperative that government agencies are consistent in reporting of this data. Consistent with this argument, federal policies have targeted improvements of epidemiological surveillance and reporting, yet there has not been a widespread check on the quality of COVID-19 data reporting across state and national levels in the U.S. The current study aims to address this gap.

The current study describes the quality of COVID-19 epidemiological surveillance data in the U.S. between July and August of 2020, in addition to re-assessing data quality at 19 months follow-up in February 2022 for five states selected as a representative subsample. Given the lack of previous literature examining the quality of public COVID-19 data that could inform directional hypotheses, this was an exploratory examination.

## Methods

### Procedures

Data were extracted from all 50 States, the District of Columbia (D.C.), and the CDC. State and district level data were extracted from individual health department COVID-19 webpages hosted by each state and D.C. Centers for Disease Control data were extracted from the CDC’s COVID Data Tracker. Data were collected and assessed on five quality dimensions. To ensure project fidelity, four research assistants (RAs) were trained on data extraction methods by project authors. This training included multiple choice, short answer, and upload fields to measure data quality dimensions. RAs then independently extracted data from health department COVID-19 websites between July 6^th^, 2020, and September 21^st^, 2020. These procedures were completed again 19 months later during February 2022 to examine for quality differences among a subset of 5 states selected based on their geographic dispersion throughout the northeastern, southeastern, central, central northern, and southwestern regions of the continental US. Availability of trained raters for follow-up assessment allowed for examination of a 5 state sample from the original total 50 states and D.C. The decision to re-examine data quality 19 months after baseline was due to rapidly changing public policies around disease prevention efforts (e.g., masking, vaccination) during that period, which were influenced by state-published COVID-19 data.

### Data sources

The extracted data were supplied by each state’s health department COVID-19 webpage and the CDC’s “Covid Data Tracker” website (Table 1). The format of these webpages varied between most states, but the essential core features included publicly available online information regarding COVID-19 prevalence and impacts within the respective state. All data were extracted from these websites using the interactive dashboards and state “data reports.”

**Table 1:** Data reported for the *Timeliness*, *Reliability*, *Accuracy*, and *Accessibility* constructs.

State	Timeliness		Reliability		Accuracy			Accessibility	
	Date of state’s earliest data report^a^	Data updated every 24 hours^b^	Identifiable source of state’s data	Source(s) of state-reported data provided independent verification^c^	Subjectively rated “oddities” present among state’s data^d^	State’s case count consistent between same-day state and CDC reports	State’s death count consistent between same-day state and CDC reports	Publicly available codebook with data definitions	Public downloadable dataset(s)
Alabama	3/11/2020	Yes	Yes	No	No	No	No	Yes	Yes
Alaska	3/11/2020	Yes	No	No	No	No	Yes	Yes	Yes
Arizona	1/22/2020	Yes	Yes	No	Yes	No	No	Yes	No
Arkansas	3/10/2020	Yes	No	No	No	Yes	Yes	Yes	No
California	3/19/2020	Yes	No	No	No	No	No	Yes	Yes
Colorado	2/29/2020	Yes	No	No	No	No	No	Yes	Yes
Connecticut	4/20/2020	Yes	No	No	No	No	Yes	Yes	Yes
Delaware	12/29/2019	Yes	No	No	No	No	No	Yes	Yes
D.C.	3/7/2020	Yes	No	No	No	No	No	Yes	Yes
Florida	3/2/2020	Yes	No	No	No	No	No	Yes	Yes
Georgia	2/15/2020	Yes	No	No	No	No	No	Yes	Yes
Hawaii	3/5/2020	Yes	No	No	No	No	Yes	No	No
Idaho	2/23/2020	Yes	No	No	No	No	No	Yes	Yes
Illinois	3/10/2020	Yes	No	No	No	No	No	Yes	No
Indiana	2/26/2020	Yes	No	No	No	No	No	Yes	Yes
Iowa	3/2/2020	Yes	No	No	No	No	No	No	No
Kansas	2/8/2020	Yes	No	No	No	No	No	No	Yes
Kentucky	3/4/2020	Yes	No	No	No	No	No	No	No
Louisiana	3/1/2020	Yes	No	No	No	No	No	Yes	Yes
Maine	3/12/2020	Yes	No	No	No	Yes	Yes	Yes	Yes
Maryland	3/23/2020	Yes	No	No	No	Yes	Yes	No	Yes
Massachusetts	3/9/2020	Yes	Yes	No	No	Yes	Yes	Yes	Yes
Michigan	3/1/2020	Yes	Yes	No	No	No	No	Yes	Yes
Minnesota	3/5/2020	Yes	No	No	No	No	No	No	Yes
Mississippi	2/1/2020	Yes	No	No	No	No	No	Yes	Yes
Missouri	2/2/2020	Yes	Yes	No	No	No	No	Yes	No
Montana	3/1/2020	Yes	Yes	No	No	No	Yes	Yes	No
Nebraska	3/6/2020	Yes	Yes	No	Yes	Yes	Yes	No	No
Nevada	3/4/2020	Yes	Yes	No	No	No	No	Yes	No
New Hampshire	2/27/2020	Yes	No	No	No	No	No	Yes	Yes
New Jersey	2/15/2020	Yes	No	No	No	No	No	No	Yes
New Mexico	4/10/2020	Yes	No	No	No	No	No	No	No
New York	3/2/2020	Yes	No	No	No	No	No	Yes	No
North Carolina	3/2/2020	Yes	Yes	No	No	No	No	Yes	Yes
North Dakota	3/11/2020	Yes	No	No	No	Yes	Yes	Yes	No
Ohio	1/2/2020	Yes	Yes	No	No	No	No	Yes	Yes
Oklahoma	3/1/2020	Yes	Yes	No	No	No	No	Yes	Yes
Oregon	1/26/2020	Yes	Yes	No	No	No	No	Yes	Yes
Pennsylvania	3/3/2020	Yes	Yes	No	No	No	Yes	Yes	Yes
Rhode Island	2/27/2020	Yes	Yes	No	No	No	No	Yes	Yes
South Carolina	3/4/2020	Yes	Yes	No	No	No	No	Yes	No
South Dakota	3/10/2020	Yes	No	No	No	No	Yes	Yes	No
Tennessee	9/29/2019	Yes	No	No	No	No	No	Yes	Yes
Texas	3/4/2020	Yes	Yes	No	No	No	No	Yes	No
Utah	3/6/2020	Yes	Yes	No	No	No	No	Yes	No
Vermont	3/8/2020	Yes	Yes	No	No	No	Yes	Yes	Yes
Virginia	2/16/2020	Yes	Yes	No	No	Yes	Yes	Yes	Yes
Washington	9/29/2019 *	Yes	No	No	No	Yes	Yes	Yes	Yes
West Virginia	3/17/2020	Yes	Yes	No	No	No	Yes	Yes	Yes
Wisconsin	3/1/2020	Yes	Yes	No	No	No	No	Yes	Yes
Wyoming	2/1/2020	Yes	Yes	No	No	No	No	Yes	Yes

^a^Date of state’s first data report at official website.

^b^Verification of whether states’ data dashboards were updated every 24 hours during July, 2020 through August, 2020.

^c^Verification was on outside external website, “provides evidence of independent verification”.

^d^Validity of the day-to-day data is questionable (i.e., rapid changes in reported numbers that don’t seem entirely plausible).

*Retrospective report.

### Interrater verification

To ensure interrater reliability, data entries were compared between RAs. The study team reconciled any inconsistencies by revisiting the respective state’s health department website and the CDC downloadable.csv file. Before proceeding to the project’s analyses, the study team established 100% agreement among the data collected.

### Measures

Five dimensions of data quality were assessed: dataset dynamicity/timeliness, trust/reliability, contextual/completeness, intrinsic/accuracy, and accessibility/accessing the data. Given the open source nature of COVID-19 data on U.S. health department websites, these quality constructs were adapted from previous literature examining the quality of open data [11] and public health information systems [10]. See Table 2 for respective indicators and measurements for the five data quality constructs.

**Table 2:** Respective indicators and measurements for each of the five data quality constructs.

Data quality construct	Measurement
*Dataset Dynamicity/Timeliness*	
Date of first reported data	Date (mm/dd/yyyy)
Updated within a month	Binary (yes/ no)
*Trust/Reliability*	
Indicated data source(s)	Binary (yes/ no)
Verification from data source(s)	Binary (yes/ no)
*Contextual/Completeness*	
All 10 “gold standard” CDC variables reported	Binary (yes/ no)
*Intrinsic/Accuracy*	
Subjective holistic assessment	Binary (complete/ not complete)
State-CDC agreement on cases & deaths	Numeric difference (e.g., Agreement: State # - CDC # = 0)
*Accessibility*	
Codebook available	Binary (yes/ no)
Downloadable dataset available	Binary (yes/ no)

### Data analysis

Data were extracted using Qualtrics surveys that were designed by the project authors. Data were downloaded, cleaned, and frequencies were calculated for each of the constructs (“Yes” =  1, “No” =  0).

### Dataset dynamicity: timeliness

Dataset dynamicity, the first quality dimension, measures whether data is “timely” and updated continuously. Zaveri et al. [9] define timeliness as, “the time point at which the data is actually used. This can be interpreted as whether the information is available in time to be useful.” To measure this construct, RAs found the first date health departments began reporting COVID-19 data to their constituents. In addition, RAs examined the frequency of data updates.

### Trust: reliability

Trust refers to examining the reliability of the data [11]. Within this dimension, the data’s reputation is assessed to determine “the integrity of a source” [11]. For this project, RAs extracted information regarding whether a state’s COVID-19 data dashboard conveyed the source(s) of reported data and whether named data sources externally verified health departments’ published data.

### Contextual: completeness of data

Contextual dimensions are defined as, “those that highly depend on the context of the task at hand as well as on the subjective preferences of the data consumer” [11]. This project examined the data completeness with an emphasis on understanding the degree of sameness between the state health departments’ reported variables as compared to the CDC. RAs examined each health department webpage for ten “gold standard” variables defined by the CDC (total cases, cases in the last 7 days, cases per 100,000 people, total deaths, deaths per 100,000 people, race/ethnicity categories [7 categories total], age [ten age categories total], sex, positive COVID-19 tests, and ICU beds occupied by all patients) and indicated whether variables were present.

### Intrinsic: accuracy

The intrinsic dimension is defined as, “whether the information correctly represents the real world and whether information is logically consistent in itself” [11]. Within this dimension, accuracy was assessed to determine if data on the COVID-19 sources were without apparent errors. RAs were trained to read COVID-19 dashboards and viewed the graphs holistically. RAs noted inconsistent reporting practices, in addition to collecting total number of cases and deaths for each state on a certain day. The RAs then consulted the CDC website and reported the total number of cases and deaths for that state on the same day. Data were analyzed to determine if reported totals were discrepant.

### Accessibility: accessing data

Accessibility dimensions determine how easily the data can be used from a researcher and/or a citizen scientist [11]. Within this dimension, availability was assessed to determine if websites contained COVID-19 datasets for public use. RAs documented whether state health department websites included a downloadable dataset and data codebook. Provision of codebooks was defined as inclusion of graph footnotes explaining abbreviations or availability of a downloadable file on the website containing definitions.

## Results

**Fig 1:**
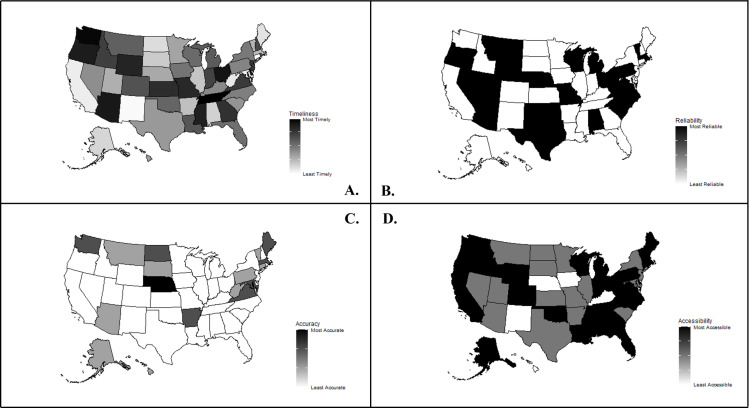
Visualization of timeliness (A), reliability (B), accuracy (C), and accessibility (D) of states’ COVID-19 data surveillance. Fig 1 Panels A–D were generated in R software [12] using the ‘usmap’ package [13]. The underlying map structure is a shapefile derived from the US Census Bureau’s TIGER/Line Shapefiles which have no copyright placed on them [14].

The quality of COVID-19 data displayed on each U.S. state and the District of Columbia’s Health Department websites was overtly poor. Of the five dimensions assessed, the most lacking dimensions were consistency, completeness, and transparency. The five data quality constructs assessed and the results for each are in the proceeding paragraphs (see Table 1) and visualized in U.S. maps (Fig 1).

### Dataset dynamicity: timeliness

Results indicated states did not collect or disseminate COVID-19 outcomes at the same time; most states (n = 30; 58.8%) began reporting data publicly in March 2020 (Range: September 2019–April 2020). Tennessee, Delaware, and Washington were the first states to report COVID-19 data, whereas Connecticut and New Mexico were the last. Despite this, all state dashboards were reported to be updated every 24 hours.

### Trust: reliability

Most data dashboards (n =  29; 56.8%) did not identify sources of published data, such as COVID-19 testing sites or hospital systems. Some states without reported data sources included California, Louisiana, and Florida. Among dashboards that conveyed the sources of reported data (n =  22; 43.1%; e.g., Arizona, Nevada, Michigan), none of the listed data sources provided external verification of the information reported by the states’ data dashboards.

### Contextual: completeness of data

Ten variables (total cases, cases in the last 7 days, cases per 100,000 people, total deaths, deaths per 100,000 people, race/ethnicity categories (7 categories total), age (ten age categories total), sex, positive tests, and ICU beds occupied by all patient) from the CDC data dashboard were assessed on each of the state health department websites. No state collected these 10 variables verbatim. For example, although most states disseminated information about age, the intervals of ages reported were not consistent with the CDC. This also occurred with race/ethnicity and gender (see Table 3 for a summary of reports).

**Table 3:** Data reported for the *Completeness* data quality construct.

CDC Standard Variable Name	Number of States Reporting Same Variable	Number of States Reporting a Similar Variable	Number of States that did not Report this Variable
*Demographics*			
Race/Ethnicity Categories	0 (0%)	49 (96%)	2 (3.9%)
Age	0 (0%)	50 (98%)	1 (1.9%)
Sex	0 (0%)	47 (92.1%)	4 (7.8%)
*Outcomes*			
Total Cases	38 (74.5%)	10 (19.6%)	3 (5.9%)
Cases in the last 7 days	1 (.01%)	28 (54.9%)	22 (43.1%)
Cases per 100,000 people	6 (11.7%)	8 (15.7%)	37 (72.5%)
Total Deaths	37 (72.5%)	14 (27.5%)	0 (0%)
Deaths per 100,000 people	4 (.07%)	3 (5.9%)	44 (86.2%)
Positive Tests	12 (23.5%)	25(49%)	14 (27.5%)
*Indicator of severity*			
ICU beds occupied by all patients	8 (15.6%)	14 (27.5%)	29 (56.9%)

### Intrinsic: accuracy

Two states’ dashboards (Arizona and Nebraska) contained an oddity in their data presented. An example of an oddity could have been the reported totals were zero when previously there were cases or deaths reported. In examining consistency between the CDC and state health department websites, most comparisons were heterogenous in the same-day reports of total number of cases (n = 43; 84.3%) and the total number of deaths (n = 35; 68.6%) attributed to each state.

### Accessibility: accessing data

A majority (n = 42; 82.3%) of dashboard websites included a downloadable codebook or graph(s) on the health department’s website with footnote documentation of abbreviations used. In addition, 34 (66.6%) states had a downloadable data set for public consumption via an unrestricted Excel file. Examples of these states include Hawaii, Idaho, Alabama, and California.

### Data quality 19 months later

In February 2022, RAs re-extracted data for New Jersey, Nevada, North Dakota, Kansas, and Mississippi following the same methods protocol. Results indicated some improvements made by health departments websites for these five states. For example, 60% states (n = 3) indicated the sources of reported data. Although, again, none of these dashboards had the information verified by the data source nor did any state utilize all 10 “gold standard” CDC variable definitions. In comparing the total number of cases for a certain day between the CDC and the state, 60% (n = 3) were consistent and 60% (n = 3) reported the same number of total deaths between the CDC and the state. In addition, all states updated their data within 24 hours, had downloadable data sets, and provided a codebook for the data.

## Discussion

Official governmental epidemiological surveillance provides a critical source of information for individuals’ appraisal of their disease risk [15], which underscores the need for a wide-scale assessment of U.S. epidemiological surveillance efforts in the context of the ongoing COVID-19 pandemic. The present study, an assessment of COVID-19 data reporting practices from 50 state health departments and the District of Columbia, revealed strengths of current U.S. COVID-19 epidemiological surveillance and several areas for improvement in public disease reporting.

Overall, U.S. states demonstrated admirable practices in the timeliness of reporting COVID-19 surveillance data and providing the information on publicly accessible data dashboards. All states updated COVID-19 data dashboards daily, which indicated ideal data timeliness and is imperative for accurately communicating current disease incidence in an area. Additionally, although not recorded in the current study’s results, all quality assessment raters reported that a vast majority of sampled data dashboards included visual depictions of relevant disease trends (e.g., bar graphs, dynamic chart filters), which is an element of public disease data communication that has been identified as an actionable step for improving public understanding of e-health informatics beyond information retained from formal, narrative reporting of disease outcomes [16]. Further, several states provided raw datasets for public download, which provides an additional display of transparency in states’ reporting of COVID-19 outcomes. Collectively, having states update their data dashboards daily with visual data depictions and allowing access to raw datasets are critical components of epidemiological surveillance that promote public trust and confidence in reported outcomes.

Study results also highlighted areas where epidemiological surveillance efforts require improvements, specifically in reporting complete data, providing reliable data to the public, and ensuring data accuracy. In the following section, we outline three aspirational recommendations intended to address fundamental discrepancies broadly apparent in these data that we believe are modifiable with downstream implications. Within this context, the CDC typically defines illnesses, identifies acceptable screening methods (i.e., specific tests, testing centers or conditions), and key reporting standards to aggregate data across heterogeneous sites and provide a national record of health in the U.S. The three recommendations described below are implementable as they draw upon these procedures the CDC already typically does and has capacity to enact. Details of implementation may involve other agencies such as the Federal Drug Administration (FDA) for testing or novel resources dedicated as part of a national strategy as the U.S. Congress did in the case of allocating PPE and testing kits from specific manufacturers in the early phase of the pandemic.

Recommendation 1: Establish formal guidelines to ensure states adhere to standardized disease outcome variables.

As demonstrated by the current results for the contextual dimension of data quality (completeness of data), there were frequent inconsistencies in the terminology used for COVID-19 reporting across U.S. states. This finding ultimately yields a disjointed national picture of COVID-19 risks and outcomes. For example, inconsistent categories for key variables such as age and race/ethnicity presented significant barriers to members of the public aiming to compare associated disease prevalence across states. Further, issues such as differences in age categories are important to local efforts of surveillance and distribution of finite resources to the most vulnerable populations. In the early months of the pandemic, there were well-known shortages in personal protective equipment (PPE), appointments for testing, and subsequently, rationing of take-home tests. By noting age-related discrepancies, efforts to allocate PPE and testing could be better coordinated in ways to address these challenges. In a larger, international review of epidemiological surveillance methods for COVID-19, Ibrahim [17] also named data completeness as a main limitation of countries’ efforts to publicly convey helpful disease information. To ensure consistency across reporting entities within the U.S. and reduce unnecessary confusion in interpretation of COVID-19 outcomes, formal guidelines should be established to ensure states adhere to standardized disease outcome variables. Standardization of variable labels and operational definitions will facilitate public consumption of disease data on a broader (e.g., national) level. Ju et al. [18] echoed this recommendation, specifically calling for further integration of public health communication frameworks to facilitate contextualization of disease risk beyond the micro/individual level and interpersonal promotion of engagement in health-protective behaviors in the context of the COVID-19 crisis.

Recommendation 2: Implement standard operational definitions and variables for data sharing across the CDC and state health departments.

The present study highlighted the need for improvements in the accuracy of reporting epidemiological surveillance. Upon comparing same-day death and case counts across hierarchical levels of reporting (e.g., CDC to state) data were discrepant for most states on a given day. Although this improved among the 5 states we recollected, it is important to highlight that discrepancies yields mistrust in reporting efforts and can be costly in evaluating risk of contracting COVID-19 and engaging in protective health behaviors. The existence of any discrepancies in data could be taken as a symptom of broader data fidelity challenges and used as rationale for data audits to address those deficiencies. In so doing, the tracking of illness could be more accurate and the public could then respond based on better data. Previous research has established people’s trust in governmental media of disease outcomes is a critical component of people the perception of disease risk and engaging in protective health behaviors [7,19]. More accurate day-to-day accounts of cases and mortality may modulate social exposure behaviors to yield a more timely but safer public response. Solutions to this problem can be sharing data or using the same operational definitions and variables between the two entities.

Recommendation 3: Require state health department websites to name testing sites for published data and describe the frequency of testing site updates to state health departments.

Data analysis found most data dashboards did not indicate who supplied data to the state health departments, which also eliminated the possibility of sharing how frequently external sites provided updated data. Further, among the few identified data suppliers, no agencies provided external verification of the totals reported on the data dashboards. Without transparency on who is providing the data, how often external sites are updating the data, nor verification from external data suppliers or, the public suffers from a lack of communication and transparency that may hinder the efficacy of broader pandemic responses [20,21]. Note, this includes the range of data providers from state testing agencies to coroners who collectively contribute to the public data record where variations in over and under-reporting can sway public perceptions. Independent verification of reported COVID-19 data by all external testing agencies is likely beyond the scope of current system resources. However, state health departments are capable of meaningfully improving transparency in their disease reporting by updating their own dashboards to publicly indicate the sources of their reported data and how often those agencies provide updates.

## Study limitations

This study was a singular assessment of health departments’ performance. Repeated sampling for all states would have provided an aggregate measure of performance. This is critical as state health departments may have evolved in how they disseminated public health surveillance data. Second, data dashboards represent an important source of disease information, but not an exclusive form. U.S. citizens also access local and national COVID-19 data through news coverage, social media sharing, additional health agencies/institutions, and additional communication from health departments (beyond dashboards). Therefore, the present study assessed a portion, but not the entirety, of COVID-19 epidemiological surveillance across U.S. states. Third, this study examined COVID-19 surveillance data at the state level, however there may be differences in reporting by counties within a state which could be further investigated in another study.

## Future directions

Future work should continue to investigate the impact of consuming surveillance outcomes on people’s engagement in COVID-19 protective behaviors. In addition, understanding where the public is consuming their knowledge and the effects of different sources is also needed. Lastly, the evidence from this paper calls for the state and federal level to best prepare for future pandemics by adopting best practices for disseminating disease information.
